# Evaluation of a systematic career coaching program for medical students in Korea using the Career Readiness Inventory

**DOI:** 10.3352/jeehp.2018.15.10

**Published:** 2018-04-18

**Authors:** Yera Hur, A Ra Cho, Eun Ji Song, Sun Kim

**Affiliations:** 1 Department of Medical Education, Konyang University College of Medicine, Daejeon, Korea; 2 Department of Medical Education, College of Medicine, The Catholic University of Korea, Seoul, Korea; 3 Konyang University College of Medicine, Daejeon, Korea; Hallym University, Korea

**Keywords:** Medical education, Career choice, Professional, Mentoring, Korea

## Abstract

**Purpose:**

The purpose of this study was to implement a systematic career coaching program for medical students and to evaluate its effectiveness.

**Methods:**

First-year medical students of Konyang University College of Medicine took part in the FLEX Mentoring II: Career Coaching Program from September to December in 2016 and 2017. This program included 16 weekly sessions, comprising a total of 32 hours. The students took the Career Readiness Inventory before and after the program, as a pre- and post-test of the program. Data from 100 students were used (46 students in 2016, 54 students in 2017) for the evaluation.

**Results:**

Medical students’ career readiness pre-test was rated as medium. In particular, many students were at a low level in terms of ‘support from colleagues and peers’ (53.0%), ‘career decision’ (48.0%), and ‘efforts for job preparation’ (60.0%). After 16 sessions of a systematic career coaching program, their career readiness level showed a significant increase except for ‘career decision’ (t= 4.242, P= 0.001) and ‘independence’ (t= 0.731, P= 0.466), a sub-factor of ‘career maturity.’

**Conclusion:**

The career readiness level of medical students was not sufficiently high. However, a semester of educational training in a systematic career coaching program helped the students to be better prepared for their career. In particular, the significant reduction in the ‘career decision’ variable after the program can be interpreted as indicating that the students changed their behavior to explore and approach their career more seriously and carefully, which also underscores the need for the implementation of career coaching programs in medical schools.

## Introduction

The need for career guidance in medical education is not only about the student him/herself, but can be also considered a social problem. Proper career decision leads health care providers to be personally satisfied with the quality of their work life and helps them optimize their performance, which eventually improves the quality of health care. This demonstrates the importance of career guidance in medical education. However, no systematic career coaching program (SCCP) has yet been developed for medical students [1]. In the past, many medical schools have planned career development initiatives for students in which faculty advisors are assigned to individual students. One-on-one career counseling initiatives have been reported to lead to significant levels of unmet needs [2]. Therefore, we developed a SCCP for medical students in each academic year based on the career coaching model [3,4]. In this study, we applied an SCCP to career guidance in medical education as a pilot course and examined the effect of the program by analyzing changes in career readiness level. Career readiness refers to an individual’s competence in managing the problems related to his or her career in each developmental stage. The subject of this research can be specified as follows: (1) What are the levels and characteristics of career readiness among first-year undergraduate medical students?; (2) How does a career coaching program change the level of career readiness?

These two overarching themes will be used as a framework for verifying the relevance and effect of our career coaching program, SCCP.

## Methods

### Ethical statement

This study was approved by the Institutional Review Board of Konyang University College of Medicine after receiving informed consent from the subjects (IRB approval no., KYUH 2014-03-014-003).

### Study design

A survey-based observational study was conducted among the undergraduate medical students of Konyang University College of Medicine using the Career Readiness Inventory (CRI).

### Subjects

First-year medical students from September to December in 2016 and 2017 of Konyang University College of Medicine participated in our newly developed systemic career coaching program called the ‘FLEX Mentoring II: Career Coaching Course.’ There were 56 and 60 students in 2016 and 2017, respectively. Among them, we analyzed data from 100 students, including 54 (male= 24, female=30) and 46 (male= 26, female= 20) students from each year, respectively. The course contained 16 weekly sessions, for a total of 32 hours. The students took the CRI before and after the program as a preand post-evaluation of the program.

### Career Readiness Inventory

The effects of the career coaching program were examined in terms of the level of career readiness. The CRI for college students assesses general developmental characteristics related to their preparation for employment. It also measures the level of employment preparation behaviors from various angles using each sub-factor and provides an objective view of career readiness [5]. This inventory is administered by the Korean Ministry of Employment and Labor and Korean Employment Information Service, and can be filled out online in Worknet, a Korean employment information website [6]. The CRI usually takes about 20 minutes, but there is no time limitation. It is divided into 2 major sections: career development (Section 1) and employment preparation behavior (Section 2). The sections have 48 and 40 questions, respectively, for a total of 88 questions (Appendix 1). Participants mark their answers to each question on a 6-point Likert scale (strongly disagree, disagree, somewhat disagree, somewhat agree, agree, strongly agree). The results of the inventory were interpreted using T scores, which were obtained by converting the raw scores for an appropriate comparison. The mean score is 50, and the standard variation is 10. The medium level is defined as T scores ranging from 41 to 60, while the low level corresponds to scores of 40 or less, and the high level is defined as scores of 60 and above. As the reliability coefficients of the sub-factors were 0.87 (career maturity), 0.92 (career search behavior), 0.74 (career decision), and 0.96 (employment preparation behavior), the item internal consistency (Cronbach alpha) of the CRI was determined to be high. Additionally, a correlation analysis showed statistically significant results, which verified the validity of the inventory. The specific details of the CRI are described in Supplement 1, and the raw data of this study are shown in Supplement 2.

### Technical information

The FLEX Mentoring II: Career Coaching Course was run from September 5 to December 12 in both 2016 and 2017. There were 16 weekly 2-hour sessions in the course. It was a required course for every student at Konyang University College of Medicine and was graded as pass or non-pass. To characterize the effects of the career coaching program, we performed a pre- and post-test of CRI before and after the program. The pre-test was done after the first session, and a full explanation of the purpose and methods of this study was provided to students in writing and orally. The post-test was done after the end of program (16th session) with the same tool.

### Statistics

The quantitative data collected from this study were analyzed using IBM SPSS ver. 21.0 (IBM Corp., Armonk, NY, USA). First, the level of career readiness was verified using descriptive statistics and cross-correlation analyses. Second, differences in the career readiness level between male and female students were assessed via the t-test for independent samples. Third, the effects of the career coaching program were analyzed in terms of differences between the pre- and post-test CRI scores through the t-test for matching samples. All results were verified by 2-tailed tests, and P-values < 0.05 were interpreted as indicating statistical significance.

## Results

### Career readiness level: pre-test

Before implementing the SCCP, all the T scores of each sub-factor of CRI were at a medium level (Table 1). However, students showed low T scores for ‘support from colleagues and peers’ (53.0%), ‘career decision’ (48.0%), and ‘employment preparation behavior’ (60.0%).

### Career readiness level: post-test after systematic career coaching program

The SCCP, titled ‘FLEX Mentoring II: Career Coaching Course,’ was a compulsory course for first-year medical students to help them experience a more practical and specific process of career exploration. It was based on our previous study and carried out as shown in Table 2. In Session 1, students received a class on the concepts of career and job and the importance of their career decision. Next, career decision and career readiness levels were measured in a counseling program in Session 2 and 3. This verified the baseline career readiness/ preparation level of the medical students, and we used an interpersonal relations inventory, a vocational value searching activity, and a psycho-geometry personality test to help them to understand themselves and others and to set directions for their path.

In the Bridge component of the program (Sessions 4–7), there were classes about strategies for exploring career information. Afterwards, students identified candidate careers using data collected from senior mentoring, career pedigree, and searching work/job list activities. Groups were then organized by similar career path and took part in a speed dating program. Speed dating is a rapid information interviewing program in which students obtain specific career information through interviewing professionals in their field of interest. It uses a semi-structured interview sheet including job specifications, work environment, required character traits, mandatory competencies, education and training, future prospects, the timing and rationale of the career decision stage, and career contentment.

The following Educational component of the program (Session 8 and 9) consisted of classes on career planning, decision making, and SMART (specific, measurable, attainable, relevant, and timely) goal setting. These activities helped the students to make a ‘career goal tree’ and to establish practical and realistic career goals. Students also took a vocational aptitude test and learned the required capabilities to make their way into their field of interest.

The next 3 sessions, Sessions 10–12, were the mentoring component of the program, which helped students specify their career goals through mentoring with academic advisors. Furthermore, each group of students presented their portfolio from the career coaching program and shared all the information they gathered. This evaluation program was run from Sessions 13–15, and the students received written feedback on their presentations from their colleagues as well as from the professor. Finally, a post-test of career readiness was conducted in Session 16 to evaluate the effects of our career coaching course.

**Fig. 1.**
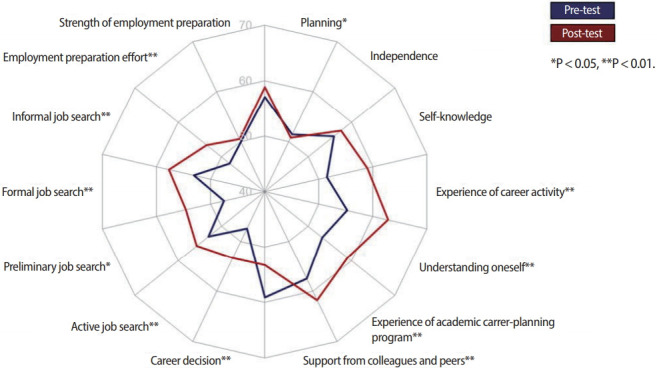
Differences in career readiness levels between the pre- and post-test T scores.

Significant differences were found in many of the factors of the CRI (Fig. 1). In particular, all the sub-factors in the ‘career search behavior’ category showed significant and positive differences before and after the SCCP. Also, 6 of the 7 sub-factors in the ‘employment preparation behavior’ category showed significant and positive differences before and after the SCCP. In contrast, it was very interesting to see students’ ‘career decision’ level drop instead of rising after the SCCP (pre-test=59.13, post-test= 53.26, P= 0.001). As shown in Fig. 1, the other survey item for which students’ post-survey score dropped after the course was ‘independence’ in the ‘career maturity’ category (pre-test= 51.42, post-test= 50.73, P= 0.466). The full results are available in Supplement 3.

## Discussion

In this study, the results of the pre-test carried out before the SCCP showed that the level of career readiness of the medical students was medium. Moreover, the score for ‘independence,’ a sub-factor of ‘career maturity,’ was the lowest; this variable assesses the degree to which individuals explore options autonomously and make their own decisions about career preparation. This demonstrates that many students rely on other people, such as parents, to make decisions about their career. In other words, even though their own career is at stake, they try to shift their responsibilities onto others. Furthermore, the scores for ‘experience of career activity’ and ‘experience of an academic career-planning program’ were the lowest in the category of ‘career search behavior.’ This indicates a deficiency in experiences of career exploration and career decision.

Many Korean medical schools have been struggling with a significant problem in career guidance because of the lack of self-exploration by students on an individual level and the presence of insufficient career information. Thus, more opportunities for those experiences should be provided to medical students in the future. Strikingly, the score for ‘support from colleagues and peers’ was the lowest among all the sub-factors. This demonstrates insufficient human support from colleagues and peers who could help students explore their future career. Moreover, many students showed a low level of career readiness in terms of ‘career decision.’ This demonstrates that medical students try to put off choosing a career because of uncertainties about their careers and future paths. In addition, ‘employment preparation behavior’ measured the level of practical performance based on the 3 items in the corresponding CRI sub-inventory.

However, since we analyzed students in their freshman year who are not at the immediate time of choosing a career, the results should be interpreted properly. Therefore, considering the career readiness of medical students as shown on the pre-test, our career coaching program (SCCP) seems appropriate as a way to help students take initiative in exploring various aspects of career information in order to develop their own career path plan. Since the SCCP helped the students understand their capabilities to deal with their career problems, it provided not only self-understanding but also an analysis of factors that could influence their choice of career.

This study proved that our coaching program (SCCP) had significant effects through the results of the post-test. Almost every item of the CRI showed a statistically significant increase. In particular, the sub-factor ‘support from colleagues and peers’ within ‘career search behavior’ showed a highly significant change from a low level— among the overall scores—to a high level. This demonstrates that the activities carried out in the SCCP, including ‘faculty and senior career mentoring’ and ‘speed-dating with professionals’ in their field of interest, served as a meaningful and valuable tool.

However, in contrast with the previous study of Yoo et al. [7], the ‘career decision’ score significantly decreased after the program. This difference can be explained in terms of the different characteristics of each subject group. In the study of Yoo et al. [7], the participants in the career coaching course were students in academic year 2 in a post-graduate medical school. Although their study used a different evaluation tool, the sub-factor ‘career decision’ in ‘career maturity’ improved compared to the control group. The students in that study were in a post-graduate medical school, meaning that they had already graduated college and re-entered medical school to become a clinical doctor. Thus, it is quite predictable that the direction of their career path would be clearer than the case for our subjects, who were undergraduate students in their freshman year in medical college. Therefore, we suggest that the decrease in the ‘career decision’ score after SCCP in our study may have been due to the characteristics of students who entered medical college based on good grades without sufficient career exploration. They experienced a systematic career exploration program, which led them to consider various career paths and become cautious about their own career decisions. This also indicates that the program accomplished its goal of encouraging students to take initiative in exploring their career and to acquire a positive and active attitude.

Medical education has long been in need of career guidance programs for medical students. For that reason, some countries have recommended structuring and formalizing a career advising system by including it in accreditation standards and national guidelines, and some medical schools are attempting to integrate a career advising system into the formal curriculum [8]. Our study documents a successful case of integrating a career coaching system into the formal curriculum for medical students.

There are some limitations in our study. First, the SCCP and CRI were only conducted in the first year of medical college, making it difficult to generalize our results to encompass career guidance throughout the scope of medical education. Second, the effects of a career coaching program can be assessed via various frameworks, such as career decision self-efficacy and career maturity, whereas we only evaluated the career readiness level. However, we have developed a SCCP [3] and suggested more specific programs for each grade [4]. To conclude, we confirmed the effect of our SCCP by running a 1-semester program in a medical college as a follow-up study. Therefore, a wider range of career coaching programs should be developed and it is expected that studies about experiences in applying such programs will be derived from our research.

## Figures and Tables

**Table 1.** The results of the pre-test for career readiness assessment

Career Readiness Inventory items	Gender		t-value	P-value	Level of T score (%)		
	Male	Female			High (T≥61)	Medium (41≤T<60)	Low (T<40)
Career development							
Career maturity							
Planning	58.52 ± 8.80	55.54 ± 8.46	1.727	0.087	33.0	65.0	2.0
Independence	52.66 ± 7.57	50.18 ± 10.05	1.394	0.167	12.0	80.0	8.0
Self-knowledge	56.60 ± 8.96	55.42 ± 8.68	0.669	0.505	25.0	70.0	5.0
Career search behavior							
Experience of career activity	54.14 ± 12.42	48.82 ± 10.74	2.291	0.024	22.0	59.0	19.0
Understanding oneself	57.24 ± 12.70	53.30 ± 11.27	1.641	0.104	34.0	56.0	10.0
Experience of an academic career-planning program	53.72 ± 9.95	52.78 ± 10.49	0.460	0.647	26.0	59.0	15.0
Support from colleagues and peers	57.18 ± 10.57	57.68 ± 9.20	-0.252	0.801	43.0	4.0	53.0
Career decision	59.06 ± 14.77	59.20 ± 10.01	-0.055	0.956	44.0	8.0	48.0
Employment preparation behavior							
Active job search	49.56 ± 13.51	45.22 ± 8.24	1.939	0.056	10.0	57.0	33.0
Preliminary job search	52.96 ± 11.36	53.10 ± 9.94	-0.066	0.948	24.0	66.0	10.0
Formal job search	49.54 ± 14.43	45.62 ± 8.24	1.478	0.143	17.0	46.0	37.0
Informal job search	53.58 ± 13.38	52.62 ± 10.06	0.405	0.686	23.0	63.0	14.0
Employment preparation effort	49.68 ± 12.48	46.44 ± 10.15	1.424	0.158	14.0	26.0	60.0
Strength of employment preparation	51.12 ± 14.01	48.64 ± 10.93	0.987	0.326	16.0	67.0	17.0

Values are presented as mean±standard deviation, unless otherwise stated.

**Table 2.** FLEX Mentoring II: Career Coaching Course

Division			Content	
Goal of the course			1.	Students perform tests and curricular activities as part of 4 types of coaching that help self-understanding in terms of character, interests, abilities, and values.
			2.	Students set their vision and purpose and complete a lifelong roadmap via a mentoring program with academic advisors.
			3.	Students set their short-term goals for career coaching during the frst semester of academic year 1 in medical school and carry out projects to explore and collect information on their career of interests
Instructional method			Types of courses: team-teaching, workshops, lectures (L).	
			Types of active learning: individual activity (IA), group/pair discussion (TA), team-based learning, presentation, quiz, interviews, group project, jigsaw, online and offline tests.	
Assessment (%)			1.	Attendance (20%)
			2.	Presentation (30%): individual/group presentation (assessed based on presentation skill and submitted materials in terms of orig- inality, organization of the content and structure, delivery skills, and teamwork), peer assessment, professor assessment
			3.	Report (50%): individual report, group report (including mentoring portfolio)
Session				
	1	Orientation	Career concept, the importance of deciding upon a career, strategies for career coaching (L); pre-test of CRI, investigation of prefer- able majors (IA)	
	2	Counseling program	Understanding of‘self’and others (1): interpersonal relations workshop (TA), instructions for using‘me’(IA+TA)	
	3		Understanding of‘self’and others (2): validation of positive personality traits, my success story, my bucket-list (IA+TA)	
	4		Understanding of‘self’and others (3): vocational values searching (IA), analysis of factors inﬂuencing the choice of a career (IA); work ethic: value stairs, career pedigree (IA+TA), senior mentoring (L)	
	5	Bridge program	Exploration of career information (1): strategies for exploring career information (L); making work/job list, collecting career informa- tion, exploring and comparing possible jobs (IA+TA)	
	6-7		Exploration of career information (2): speed-dating program with professionals of interest (TA)	
	8	Educational program	Career planning: vocational aptitude test (IA), career planning and decision process (L), career goal tree (IA)	
	9		Goal-setting:‘My Dream’story book (IA), SMART goal setting (IA)	
	10	Mentoring program	Formation of relationships: self-prediction (IA), strategies for communication and studying (L), reading guidance (IA)	
	11		Details of career planning: the rule of 21 days, prediction of career roadblocks, my future business card (IA)	
	12		Performance and accomplishments: career tree, lifelong map (IA)	
	13-15	Evaluation	Presentation and information sharing (TA+PA)	
	16		Evaluation of reports and feedback, post-test of the CRI	

L, lectures; IA, individual activity; TA, group/pair discussion; PA, peer assessment; CRI, Career Readiness Inventory; SMART, specific, measurable, attainable, relevant, and timely.

## Supplementary materials

Supplement 1. Contents of the career readiness inventory.

Supplement 2. Data files are available from https://doi.org/10.7910/DVN/03BW6X

Supplement 3. Differences in career readiness levels between the pre- and post-SCCP T scores.

Supplement 4. Audio recording of the abstract.

## Appendix 1. Career Readiness Inventory

**Section 1:** Career development

No.	Question	1	2	3	4	5	6
1	I have a plan to study a major/career of my interest.						
2	I have been thinking about what to do to make my dreams come true.						
3	I am very aware of what I cannot do well						
4	I am very aware of what I like to do.						
5	I am willing to choose a career in spite of my parents’objections.						
6	I am aware of what I do not like to do.						
7	I know the bad points of my personality.						
8	It is better to choose my career based on my seniors’opinions.						
9	I am willing to choose what I like to do even though it is a very difficult task.						
10	I know the good points of my personality.						
11	I usually make a plan before doing things.						
12	My thoughts are more important for making career decisions than others’opinions.						
13	I focus on the prospects of jobs when choosing one.						
14	My job decision is very influential in my future life.						
15	I am willing to choose my career according to what I want rather than based on my seniors’decision.						
16	I know what I am interested in.						
17	I have a definite plan for my career compared to my peer group.						
18	I have taken classes related to job exploration to get to know the fields of work that are suitable for me.						
19	I have researched various fields of work through classes related to job exploration.						
20	I have taken classes related to self-exploration to get to know what kind of person I am.						
21	I have found some psychological tests to know myself better.						
22	I have taken a psychological test to find an adequate career for myself.						
23	I have made a list of what I like/dislike to consider in my career decision.						
24	I have talked to more senior individuals in various fields to get information of their job fields.						
25	I have asked some more senior students of the same major about their future career or job plans after their graduation.						
26	I have asked professors about career fields or jobs for undergraduates.						
27	I have searched information to find appropriate jobs via the college’s employment information center.						
28	I have received counseling about my characteristics and psychological problems in the student counseling center of my college or from professionals of other counseling organizations.						
29	I have talked about my strengths and weaknesses or my personality with my siblings or cousins.						
30	I have taken an online psychological test to determine more precisely my aptitudes, interests, and character.						
31	I have participated in seminars or group counseling programs (for example, self-growth programs) to understand myself better.						
32	I have discussed my aptitude and future career with colleagues.						
33	I have discussed my aptitude and future career with professors.						
34	I have discussed my aptitude and future career with friends.						
35	I have discussed my aptitude and future career with my parents or relatives.						
36	I have attended various career/job presentations.						
37	I have experimented with various career activities.						
38	I have purchased or read a guidebook or brochures about educational training organizations or programs related to the job/ career of my interest in the last few weeks.						
39	I have visited the student counseling center or other counseling organizations to consult about my career problems in the last few months.						
40	I have made my decision about my career, and I am pleased with it. Also, I know what I should do to accomplish my goals.						
41	I am pleased with the major that I have chosen for myself. Also, I know what I should do to do to accomplish my goals.						
42	I believe that I am talented and I can be anything I want with the opportunities I have been given, but it is impossible in reality. Moreover, I still have not thought of any other alternatives.						
43	I am having trouble choosing a single job among those that I am equally interested in.						
44	Even though I want to be a _______ (something), I cannot make any career decision right now because of people who care about me but have different opinions. I would like to have a job corresponding with those opinions.						
45	I have not thought about my career decision much. I am confused because I have not made many decisions for myself before. In addition, I do not have enough information to choose a career right now.						
46	Everything about my career decision is too vague and uncertain, so I want to postpone making any decisions for now.						
47	I thought I knew what kind of career I want, but I recently figured out that it is impossible. Therefore, I am trying to find other possible career paths now.						
48	I want to make my career decisions fast because it is too much of a burden for me to consider these options for a long time.						

**Section 2:** Employment preparation behavior

No.	Question	1	2	3	4	5	6
49	I collect employment information from newspapers, magazines, and books.						
50	I collect recent employment information via TV advertisements.						
51	I get recent employment information via radio broadcasts or advertisements.						
52	I search and collect employment information from relevant websites.						
53	I collect employment information using the career or employment center of my college.						
54	I attend various job/career fairs and workshops.						
55	I get employment information by discussing employment with my family or relatives.						
56	I discuss my job search with my friends or get employment information from them.						
57	I discuss my job search with colleagues in the fields of my interest or get employment information from them.						
58	I get employment information from already-established professionals in my field of interest.						
59	I ask my friends for advice about my job search.						
60	I ask my colleagues and peers for help in my job search even when they are busy.						
61	I ask my family or relatives about what kind of job they want me to choose.						
62	I ask my former colleagues from my last part-time job for help with my job search.						
63	I tell my colleagues and peers that I am still unemployed and searching for jobs.						
64	I ask people whether there is anyone who can help me to get a job.						
65	I prepare specific work experiences to include in my resume.						
66	I carefully read newspaper or magazine articles that present jobs or work opportunities.						
67	I put effort into collecting as much information as possible about jobs I am considering.						
68	I read various books, promotional materials, and brochures to gather information about jobs of interest.						
69	I search online for information about fields I am interested in and organize that information.						
70	I search for information at my college’s employment center to find a suitable job.						
71	I have joined an internet café or communities to share information with people who hope for similar jobs after graduation.						
72	I prepare for some qualification certificates related to foreign language skills including English.						
73	I prepare for some qualification certificates related to computer skills.						
74	I prepare the national qualification exams related to my fields of interest.						
75	I study hard for my major or study in a group to ensure I get good grades.						
76	I take courses related to my future job experiences.						
77	I register as a job applicant in a recruiting companies or my dream company.						
78	I send my resume to a company that I want to be employed with.						
79	I visit the company and go through interviews for employment.						
80	I practice and train for a job interview.						
81	I participate in a company briefing presentation.						
82	I send my resume to a human resources manager.						
83	I fill out a job application form.						
84	I have had a job interview with a human resources manager.						
85	I have visited a recruiting company.						
86	I have called a human resources manager.						
87	How much of your time do you invest in your job/employment search per week?						
	① Less than 1 hour ② 1 hour or more, but less than 5 hours ③ 5 hours or more, but less than 10 hours						
	④ 10 hours or more, but less than 15 hours ⑤ 15 hours or more, but less than 20 hours ⑥ 20 hours or more						
88	How much effort do you think you have put into job/employment search?						
	① Never tried before ② Not tried ③ Not tried very much ④ Tried to some extent ⑤ Tried ⑥ Tried my best ever						

## References

[1] An H, Kim E, Hwang J, Lee S (2014). Analysis of medical students’ needs for development of a career guidance program. Korean J Med Educ.

[2] Sastre EA, Burke EE, Silverstein E, Kupperman A, Rymer JA, Davidson MA, Rodgers SM, Fleming AE (2010). Improvements in medical school wellness and career counseling: a comparison of one-on-one advising to an Advisory College Program. Med Teach.

[3] Hur Y (2016). Development of a career coaching model for medical students. Korean J Med Educ.

[4] Hur Y, Cho AR, Kwon M (2018). Development of a systematic career coaching program for medical students. Korean J Med Educ.

[5] Yoo GS (2014). User guide: career readiness inventory.

[6] Korea Employment Information Service Career readiness inventory [Internet]. http://www.work.go.kr/consltJobCarpa/jobPsyExam/univJobPreDetail.do.

[7] Yoo HY, Park KH, Kim SY, Im SJ (2015). The effectiveness of a career design program for medical students. Korean Med Educ Rev.

[8] Howse K, Harris J, Dalgarno N (2017). Canadian national guidelines and recommendations for integrating career advising into medical school curricula. Acad Med.

