# Supplementary material for: Evaluation of a systematic career coaching program for medical students in Korea using the Career Readiness Inventory
Source: J Educ Eval Health Prof. 2018 Apr 18;15:10. doi: 10.3352/jeehp.2018.15.10 (PMC5968220; doi:10.3352/jeehp.2018.15.10)
Supplement: Supplementary file 1 — Supplement 1. Contents of the career readiness inventory. [file jeehp-15-10-suppl.pdf]

Supplement 1. Contents of the Career Readiness Inventory

Section	Category	Sub-inventory	Content	Sub-factors (no. of items)	Content
Section 1	Career development	Career maturity	An individual's level of readiness to deal with career problems at the developmental stage	Planning (6)	The level of advance preparation and planning to make decisions on career direction and work/jobs
				Independence (5)	The level of individual readiness for career exploration, preparation, and decisions for oneself
				Self-knowledge/understanding (6)	The level of understanding of an individual's capabilities, interests, character, and values
		Career search behavior	The level of performance of cognitive and behavioral activities to understand oneself and the world of work for career decision or development	Experience of career activity (7)	The extent of experiences with activities to search for 'self-characteristics' and career information
				Understanding oneself (5)	The extent of experiences of activities to understand the individual's aptitude, interests, character, etc.
				Experience of academic career-planning program (3)	The extent of experiences of classes aiming to find suitable career paths for each individual
				Support from colleagues and peers (7)	The level of support and resources from others during the career exploration process
		Career decision	The level of assurance about career decision and the level of decision-making in the continuum between the clear career decision and the unclear pending one	Career decision (9)	The degree to which a career decision is made based on self-understanding and identification of a career of interest
Section 2	Employment preparation behavior	Employment preparation behavior	The level of behaviors to manage more realistic and specific problems related to one's career path	Active job search (10)	The level of preparatory behaviors for immediate and definite career exploration and selection process
				Preliminary job search (10)	The level of data collection related to one's career and jobs and preparing one's potential in the middle of the job search
				Formal job search (6)	The level of preparatory behavior for using formal information sources including the media, presentation meeting, internet, etc.
				Informal job search (6)	The level of preparatory behavior for using informal information sources including friends, family, senior colleagues, etc.
				Employment preparation effort (6)	The level of relative effort for employment preparation and exploration
				Strength of employment preparation (2)	The level of strength of employment preparation in terms of time and effort
