# Supplementary material for: Evaluation of a systematic career coaching program for medical students in Korea using the Career Readiness Inventory
Source: J Educ Eval Health Prof. 2018 Apr 18;15:10. doi: 10.3352/jeehp.2018.15.10 (PMC5968220; doi:10.3352/jeehp.2018.15.10)
Supplement: Supplementary file 2 — Supplement 3. Differences in career readiness levels between the pre- and post-SCCP T scores. [file jeehp-15-10-suppl3.pdf]

Supplement 3. Differences in career readiness levels between the pre- and post-test CRI scores

Career Readiness Inventory items	Pre-test	Post-test	t-value	P-value
**Career development**				
Career maturity				
Planning	57.03±8.72	58.88±9.25	-2.087	0.040
Independence	51.42±8.94	50.73±7.85	0.731	0.466
Self-knowledge	56.01±8.79	57.62±9.78	-1.599	0.113
Career search behavior				
Experience of career activity	51.48±11.86	58.99±10.85	-6.510	0.001
Understanding oneself	55.27±12.11	62.81±8.39	-6.528	0.001
Experience of academic career-planning program	53.25±10.18	59.15±8.70	-5.196	0.001
Support from colleagues and peers	57.43±9.86	61.77±8.13	-4.673	0.001
**Career decision**	59.13±12.55	53.26±14.33	4.242	0.001
**Employment preparation behavior**				
Active job search	47.39±11.34	53.29±12.48	-4.153	0.001
Preliminary job search	53.03±10.62	55.68±10.01	-2.155	0.034
Formal job search	47.58±13.34	54.55±12.50	-5.548	0.001
Informal job search	53.10±11.79	57.69±11.87	-3.908	0.001
Employment preparation effort	48.06±11.44	53.41±11.28	-4.569	0.001
Strength of employment preparation	49.88±12.56	50.45±10.55	-0.573	0.568

Values are presented as mean±standard deviation, unless otherwise stated.

CRI, Career Readiness Inventory
